# Effect of saliva on accuracy of digital dental implant transfer using two different materials of intraoral scan bodies with different exposed lengths

**DOI:** 10.1186/s12903-024-05199-1

**Published:** 2024-11-23

**Authors:** Mohamed Hesham Ahmed Tawfik, Ibrahim Ramadan EL Torky, Mohamed Maamoun El Sheikh

**Affiliations:** 1 Prosthodontic Department, Faculty of Dentistry, MUST University, Giza, Egypt; 2 Prosthodontic Department, Faculty of Dentistry, Tanta University, Tanta, Egypt

**Keywords:** Scan body, Digital transfer, Implant impression, Intraoral scanner, Saliva

## Abstract

**Background:**

The accuracy of digital implant transfer is currently under investigation in relation to the effect of saliva, scan body material, and exposed length.

**Methods:**

Six completely edentulous casts with four implant fixtures were fabricated. The four implant fixtures in each cast were placed below the crest of the ridge of the casts by 1.5 mm. The four implant fixtures were alternately attached to four implant scan bodies (PEEK) Group (I) and (TITANIUM) Group (II). For each cast, two flexible polyurethane layers with varying thicknesses were fabricated and molded on the six 3D printed identical casts after the placement of the implant fixtures in each cast for the soft tissue moulage in the cast. The six casts were alternately covered with a 2 mm and 4 mm thick layer. The six reference casts were recorded using a coordinate measurement machine (CMM) and subsequently scanned with an Intraoral scanner (MEDIT I 700). The scanning was conducted under both dry and wet conditions, with artificial saliva applied. The scanning platform consisted of two transparent acrylic boxes, and the process followed standardized scanning conditions using a digital lux meter (*n* = 48). The mean difference and standard deviation values (± SD) between the implant scan bodies measured on the reference and experimental scans were calculated using the inspection software program (Medit design). Data were fed to the computer and analyzed using IBM SPSS software package version 20.0. (Armonk, NY: IBM Corp). The Kolmogorov-Smirnov test and Shapiro-Wilk test were used to verify the normality of distribution. Quantitative data mean and standard deviation. The significance of the obtained results was determined at the 5% level. The student t-test for normally distributed quantitative variables was used to compare the two groups studied.

**Results:**

Statistically significant differences in wettability condition discrepancies were found between groups (I) and (II) (*p* < 0.05). In addition, there were statistically significant differences in intraoral scan body (ISB) length between the two groups (*p* < 0.05). Regarding the intraoral scan body material, statistically significant differences were found between the two groups (*p* < 0.05).

**Conclusions:**

The presence of saliva significantly affects the accuracy of the digital implant transfer. Additionally, using a longer intraoral scan body improves the accuracy of digital implant impressions. The titanium scan body had a greater level of precision on the implant impression scan.

## Background

The passive fit of an implant-supported framework is a critical factor in achieving successful treatment outcomes over the long term [1, 2]. Superstructure mismatches may result in biomechanics issues [3, 4]. Precision and trueness are two components of accuracy, as defined by the International Organization for Standardization (ISO) 5725-1. Precision refers to the degree of conformity between a measurement and an approved or true reference, whereas trueness defines the degree of agreement between successive measurements and the same value [5]. The lack of standardization in clinical and laboratory methods may have an adverse effect on the accuracy of the prosthesis [6]. The misalignment of the implant superstructure is due to the cumulative effect of the varying degrees of inaccuracy in these steps [7]. The accuracy of the impression is a crucial factor that significantly affects outcomes since it is the initial stage in the fabrication of restorations [8, 9].

A digital model is generated by utilizing the data collected from the scan body and its surrounding tissues. Scanning technology enables the precise digital determination of the optimal placement of implant superstructures [10, 11]. Direct digital implant impressions offer several advantages over traditional impression processes, including the reduction of chairside time and an increase in patient acceptance [12]. There is a scarcity of research regarding the impact of various liquids on the precision of intraoral scanner outcomes when used in dry conditions and on the tooth surface. Furthermore, it is important to consider the elevated levels of humidity in the oral environment. Consequently, despite the constant presence of liquids in the mouth, the means to reduce their influence on the accuracy of intraoral scanners remains unknown. There is a lack of evidence regarding the use of compressed air from a three-way syringe to remove saliva from tooth surfaces before intraoral scanning, despite manufacturers’ recommendations. Previous studies have considered or proposed that factors such as saliva, blood, gingival crevicular fluid, and the moist environment in the mouth may affect the accuracy of intraoral scanners [13]. In order to decrease scanning errors, some manufacturers of intraoral scanners suggest drying the area using compressed air before scanning it.

Nevertheless, there is presently a lack of empirical evidence to support these hypotheses. For instance, there is no substantiation that a three-way syringe adequately dehydrates samples or that liquid adversely affects scanning outcomes. To the best of our knowledge, this study represents the first investigation into the impact of an adhesive substance on the accuracy of an intraoral scanner when applied to a tooth surface. The dentistry literature lacks sufficient research on the impact of surface humidity on intraoral scanning accuracy [14]. However, the findings of those studies have been inconsistent. In a laboratory experiment, Chen et al. [14] utilized fully dentate and scanned utilizing two IOSs in either dry, moist, or air-blown circumstances (submerging and then draining). The dry condition exhibited the highest level of accuracy compared to the other groups. After choosing a partly dentate cast with three implant analogs inserted, Park et al. used an intraoral simulator to mimic two scenarios with varied temperatures and humidity levels. To find out any discrepancies in the digital images, we measured the lengths between the arches. According to the authors, ambient factors did not have any impact on scan accuracy [15]. The implant depth is crucial as it directly impacts the visibility of the body during the scan, consequently influencing its accuracy. Typically, the duration of the implant’s presence directly correlates with the required length of the scan body for optimal visibility. Arcuri et al. used digital impressions to investigate the depth impact of implants placed 3 and 6 mm subgingivally. The results indicated that the accuracy of digital impressions remained unaffected by the depth of the implant [16]. According to another study conducted by Gimenez-Gonzalez et al., the accuracy of digital impressions was enhanced by subgingivally spaced implants (2 and 4 mm subgingivally). Therefore, implant depth should be taken into account when selecting an ISB design [17]. The material of the scan bodies could also play a role. However, the only evidence available to date suggests that the data acquired by an IOS is more precise as the scanned material becomes more opaque [18]. Therefore, it appears that scanning metallic surfaces provides inaccurate findings [19]. When examining an SB, it is crucial to take into account its design, structure (whether it is composed of one or two pieces), and material (such as titanium and/or polyether-ether-ketone, with a preference for PEEK due to its optimal optical properties) [20], along with the manufacturing tolerances for the components. In a study comparing the trueness value of scan body materials, the titanium scan body demonstrated superior performance compared to the PEEK scan body. In order to prevent any potential interference, this study used PEEK and titanium scan bodies, both manufactured by the same company, to maintain the shape of the scanned body [21, 22]. We aimed to independently assess the impact of scan body materials on the reliability of scan data. The titanium and PEEK scan bodies utilized in this investigation were comparable. However, the titanium scan bodies had two flat faces. This difference may have resulted in the titanium scan bodies being more accurate in terms of RMS compared to the PEEK ones. Titanium scan bodies also demonstrated broader interquartile ranges for RMS values and a lower within-tolerance value compared to PEEK scan bodies. According to the manufacturer’s recommendations, scanning powder should not be used when scanning titanium scan bodies. Nevertheless, it was found that shiny metal objects exhibited lower scanning precision [23]. Due to the matted surface of TITANIUM scan bodies, the scan results of titanium scan bodies in the present study may be jagged. This lack of precision could potentially restrict the use of titanium scan bodies in clinical practice. **Aim of the study**: The aim of this in vitro study is to evaluate the effect of saliva on the accuracy of digital dental Implant transfer according to the exposed length of two different materials of Intraoral scan bodies.

### First null hypothesis is that

Saliva has no effect on the accuracy of digital scans obtained using (PEEK) and (TITANIUM) scan bodies.

### Second null hypothesis is that

The exposed length of scan bodies has no effect on the accuracy of digital scans.

## Methods

### Study setting

This experimental study was conducted in the Prosthodontic Department and the CAD/CAM laboratory of the Faculty of Dentistry at Tanta University.

### Study’s sample size

The sample size for this investigation was 48 scans. In this investigation, the power sample size was greater than 80%, and the significance threshold was set at 0.5. The statistical power was 80%, and the confidence interval was 95%. The statistical software SPSS 25 (IBM, Inc., Armonk, NY) was used for all analyses, and a significance level of α = 0.05 was set. The Kolmogorov-Smirnov test was used to determine homoscedasticity, and the Shapiro-Wilk test was used to determine the normality of deviations. The level of statistical significance was set at 0.5.

### Cast preparation

An epoxy resin dental prosthetic cast, designed for a toothless mandibular area, was scanned using an industrial computed tomography device called a bench scanner. This scanning process was done at Ramses Medical Products Factory in Egypt to create a master cast. This is from SEOUL, Korea (DOF freedom HD): 0479. In order to import the STL file into the dental CAD software (Exocad Dental CAD, Exocad, Darmstadt, Germany), the model was exported in Standard Tessellation Language (STL).

### Printing and cast design

**Fig. 1:**
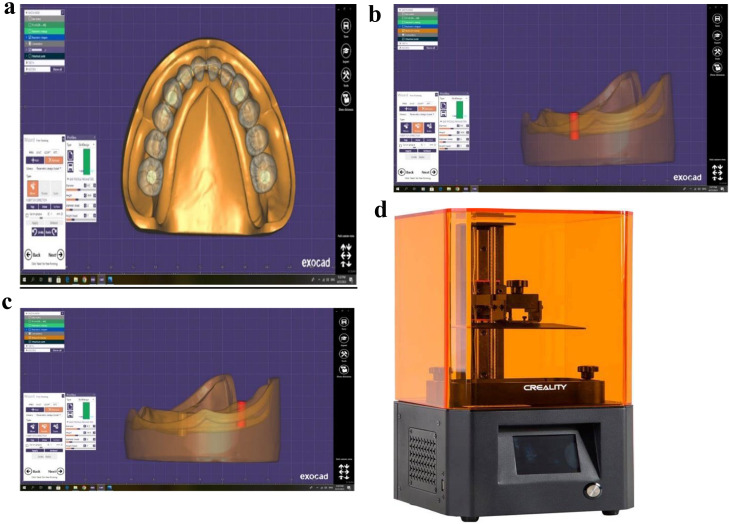
**a**: Virtual setting of teeth using EXOCAD DESIGNING SOFTWARE. **b**: Virtual implant fixtures catback using EXOCAD DESIGNING SOFTWARE. **c**: Virtual implant fixtures catback using EXOCAD DESIGNING SOFTWARE. **d**: 3D printer (CREALITY 3D printer, HALOT, UK)

Dental prosthetics were digitally fitted to the master cast from first lower molar to the other first molar on the other sideas shown in Fig. 1a. Subsequently, all of the virtual teeth were extracted, excluding the canines and first molars on both sides. This enabled to accurately allocate the four implant fixture positions at the canines and molars. Exocad design software was utilized at these specific locations to obtain cutbacks for implant fixtures measuring 4.3 mm in diameter and 10.5 mm long, as depicted in graphics 1b, c. Using a 3D printer (CREALITY 3D printer, HALOT, UK), six resin casts were created, each with the preset placements of four implant fittings. The resin was obtained from China (PROSHAPE MODEL 3D PRINTING405 NM UV RESIN), as depicted in graphics 1d, 2a.

**Fig. 2:**
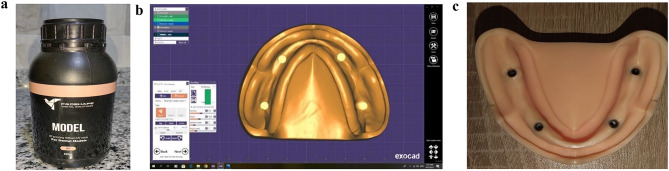
**a**: PROSHAPE MODEL 3D PRINTING405 NM UV RESIN. **b**: Virtual implant fixtures catback using EXOCAD DESIGNING SOFTWARE. **c**: Implant fixtures placed in the predetermined positions

The 3D-printed casts were washed and cured using a washing and curing machine (ANYCUBIC, China). Four implant fixtures measuring 4.3 mm in diameter and 9 mm in length (C-TECH Implants n.240123, Bologna, Italy) were placed in the right canine, right first molar, left canine, and left first molar areas. Four implant fixtures 1.5 mm had been inserted below the level of the alveolar bone for each mold, as demonstrated in Fig. 2 (b and c).

### Cast verification and checking

**Fig. 3:**
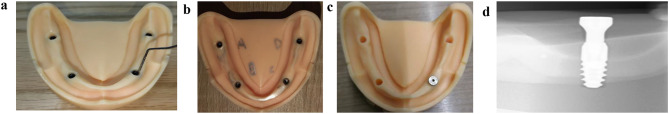
**a**: Checking the depth of implant fixtures with a graduated periodontal probe. **b**: Four implants’ positions marked in the cast. **c**: Cast with healing abutment. **d**: Periapical X-ray for the cast with healing abutment

The accuracy of the four implant fittings was examined twice using a graded periodontal probe. Four sets of implants were designated on each cast: (A) for the right first molar, (B) for the right canine, (D) for the left first molar, and (C) for the left canine. In order to prevent any rotational or vertical movements, the four healing abutments were carefully placed over the four implant fixtures in each cast using the same screwdriver provided by the implant company. In order to ensure proper placement of the implant fixings and healing abutments, periapical X-rays were obtained for each cast. The CMM (coordinate measuring machine, S/N GS0c/9131, UK) was used to measure six interimplant distances in micrometers at the center of the healing abutment as a reference value. The implant fixtures were positioned precisely at the center of the healing abutment. A coordinate measuring machine (CMM) was used to measure the distances for each of the six casts. The three-dimensional coordinates (x, y, and z) at the centers of the implant platforms were recorded. According to the manufacturer, the CMM had an accuracy of 0.0001 mm, and all measurements were taken using a single operator. In this context, “center of implant position” refers to the spot where the healing abutments, which were secured to the implant fixtures, were located. To determine the exact center of each healing abutment, a CMM probe was used to touch eight different spots around the outer diameter of the abutment. At this point, the flat surface was considered XY. The vertical distances between four healing abutments in the Z-axis were calculated by measuring four locations on the upper surface of each abutment to construct a plane. The PEEK and TITANIUM scan bodies underwent periapical X-rays to verify the precise positioning of the intraoral scan bodies, as shown in Fig. 3a–d.

### Placement of intraoral scan bodies over the implant fixtures

**Fig. 4:**
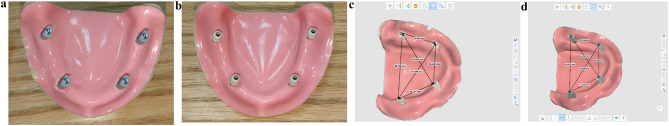
**a**: TITNIUM intraoral scan bodies. **b**: PEEK intraoral scan bodies. **c**: Measuring six interimplant distances between the PEEK intraoral scan bodies. **d**: Measuring six interimplant distances between four TITANIUM intraoral scan bodies

At the beginning. four PEEK intraoral scan bodies were screwed on the 4 implant fixtures in each cast, The exposed length of the scan bodies was regulated by covering the cast with 2 mm of flexible polyurethane layer which and did a dry scan and wet scan for the cast, then the 2 mm polyurethane layer was replaced with another layer of polyurethane with 4 mm thickness and did another dry and wet scan. These flexible polyurethane layers were fabricated, shaped and molded on each cast of the 3D printed casts after the casts were fabricated and printed with the predetermined implant positions in each cast. The previous protocol was applied on each cast of the six casts (so a total of four digital scans on the each cast using PEEK intraoral scan bodies).Then four TITANIUM intraoral scan bodies were screwed on the 4 implant fixtures in each cast, The exposed length of the scan bodies was regulated by covering the cast with 2 mm of flexible polyurethane layer and did a dry scan and wet scan for the cast, then the 2 mm polyurethane layer was replaced with another layer of polyurethane with 4 mm thickness and did another dry and wet scan. The previous protocol was applied on each cast of the six casts (so a total of four digital scans on the each cast using TITANIUM intraoral scan bodies).So the total number of scans done was 48 scans, as shown in Fig. 4a, b.

**Fig. 5:**
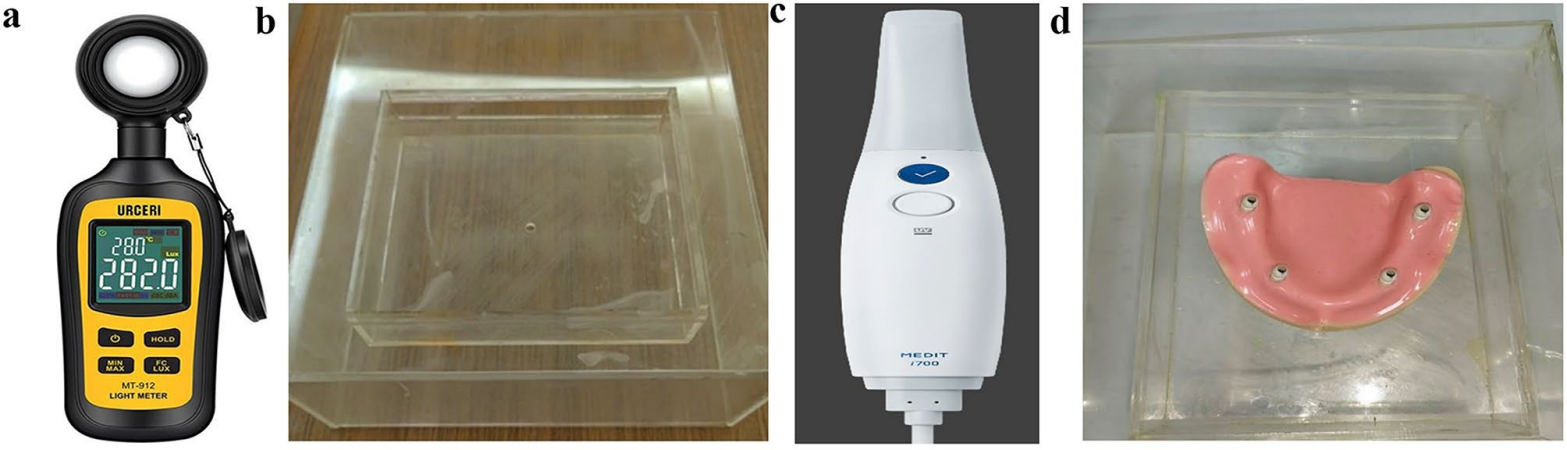
**a**: URCERI digital luxmeter (china). **b**: Two acrylic transparent boxes (scanning platform). **c**: MEDIT i700. d: tested cast affixed to the scanning platform

A digital luxmeter and a scanning platform consisting of two transparent acrylic boxes of different sizes (10 × 13 × 3 cm and 18 × 18 × 7 cm). The small rectangular box, having a 4 mm diameter hole on its bottom, was affixed to one of the upper corners of the larger box. Each tested cast was attached at the center of the bottom of the small box using utility wax were utilized to conduct 48 scans. The hole of the small box was blocked with wax and artificial saliva was slowly poured from the corner of the small box until the model was completely immersed, and the liquid reached a predetermined height (3 cm from the bottom of the smaller box to make sure complete coverage of the scan bodies), as shown in Fig. 5a, b,d.

The wax which was applied to seal the hole of the smaller box was then removed to allow the artificial saliva to drain slowly into the larger box, leaving the tested cast surface with the attached scan bodies wet with the artificial saliva [14]. Each cast was then scanned again using the same intraoral scanner. scans using an intraoral scanner (MEDIT i700, SEOUL, South Korea), as shown in Fig. 5c, in both dry and wet circumstances with artificial saliva, on each PEEK intraoral scan body there were a marked reference point fabricated on the top edge of the scan region of the scan body for the standardization of the scans done with the PEEK intraoral scan body, and for the TITANIUM intraoral scan body the center of the screw at the top of the TITANIUM intraoral scan body were taken as a marked reference point. To determine the interimplant distances between the four fixtures, the prior scans were imported into our specialized measuring program (MEDIT DESIGN, version 3.1.0). Then, these measurements were compared with the ones obtained using CMM as a reference, as demonstrated in Images 4c, d.

### Data analysis

Data from the specified measures were collected, tabulated, and analyzed using IBM SPSS software package version 20.0 (New York: IBM Corp., Armonk, 1980). The Shapiro-Wilk and Kolmogorov-Smirnov tests were used to verify the normality of data. To represent quantitative data, we utilized standard deviation and mean difference. The Student’s t-test was utilized to compare the means between all groups.

## Results

**Fig. 6:**
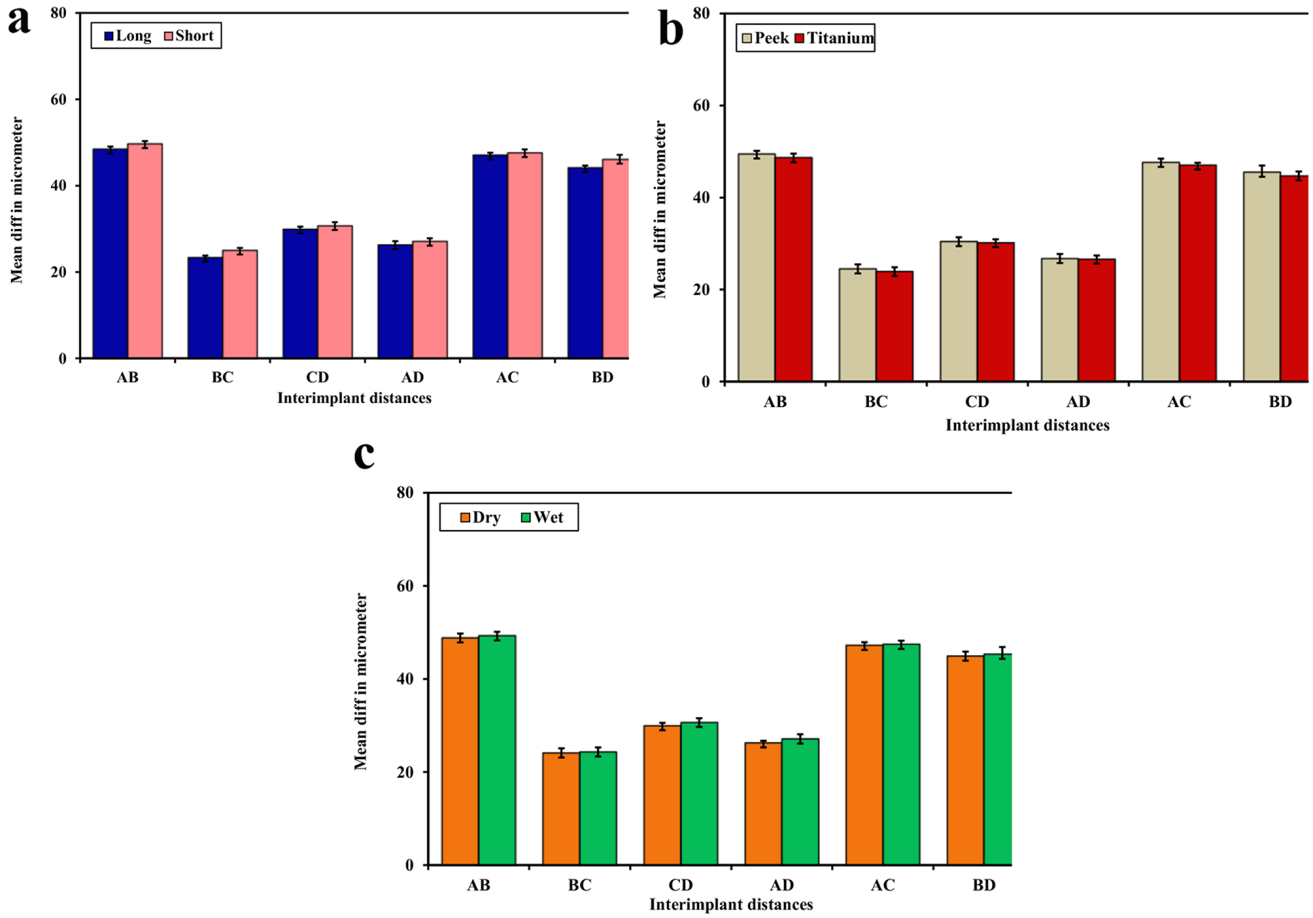
**a**: Mean difference of distances in micrometer, standard deviations for the effect of the exposed length of intraoral scan body. **b**: Mean difference of distances in micrometer, standard deviations for the effect of the material of intraoral scan body. **c**: Mean difference of distances in micrometer, standard deviations for the effect of the wettability of intraoral scan body

Table 1; Fig. 6a show the effect of the length of the intraoral scan body. There were significant differences in mean values and standard deviation between the distances (measured in micrometers) of the PEEK intraoral scan body with different exposed lengths (long and short) in six interim plant distances. (*p*-value < 0.05).

**Table 1:** Mean difference of distances in micrometer, standard deviations, *p-*value, and t-value for the effect of the exposed length of intraoral scan body

	(AB)	(BC)	(CD)	(AD)	(AC)	(BD)
**Long (L)**	48.46 ± 0.63	23.39 ± 0.40	29.93 ± 0.56	26.30 ± 0.82	47.08 ± 0.53	44.17 ± 0.49
**Short(S)**	49.70 ± 0.62	25.06 ± 0.52	30.73 ± 0.83	27.11 ± 0.71	47.63 ± 0.76	46.09 ± 1.07
**T**	6.890^*^	12.522^*^	3.894^*^	3.621^*^	2.895^*^	8.041^*^
***P***	< 0.001^*^	< 0.001^*^	< 0.001^*^	0.001^*^	0.006^*^	< 0.001^*^

24 replica. Data was expressed using Mean ± SD. SD: Standard deviation. t: Student t-test. *p*: *p*-value for comparing between Long and Short. *: Statistically significant at *p* < 0.05

Table 2; Fig. 6b show the effect of the material on the intraoral scan body. There were significant differences in the mean values and standard deviation in the distance between the materials of the intraoral scan body, PEEK, and TITANIUM in six interim plant distances (*p*-value < 0.05).

**Table 2:** Mean difference of distances in micrometer, standard deviations, *p* value and t-value for the effect of the material of intraoral scan body

	(AB)	(BC)	(CD)	(AD)	(AC)	(BD)
**Peek**	49.48 ± 0.69	24.51 ± 0.95	30.45 ± 0.90	26.76 ± 0.98	47.65 ± 0.79	45.53 ± 1.44
**Titanium**	48.68 ± 0.88	23.95 ± 0.90	30.21 ± 0.71	26.65 ± 0.74	47.07 ± 0.48	44.73 ± 0.95
**T**	3.484^*^	2.096^*^	0.995	0.433	3.066^*^	2.255^*^
***P***	0.001^*^	0.042^*^	0.325	0.667	0.004^*^	0.029^*^

24 replica. Data was expressed using Mean ± SD. SD: Standard deviation. t: Student t-test *p*: *p*-value for comparing between Peek and Titanium. *: Statistically significant at *p* < 0.05

**Fig. 7:**
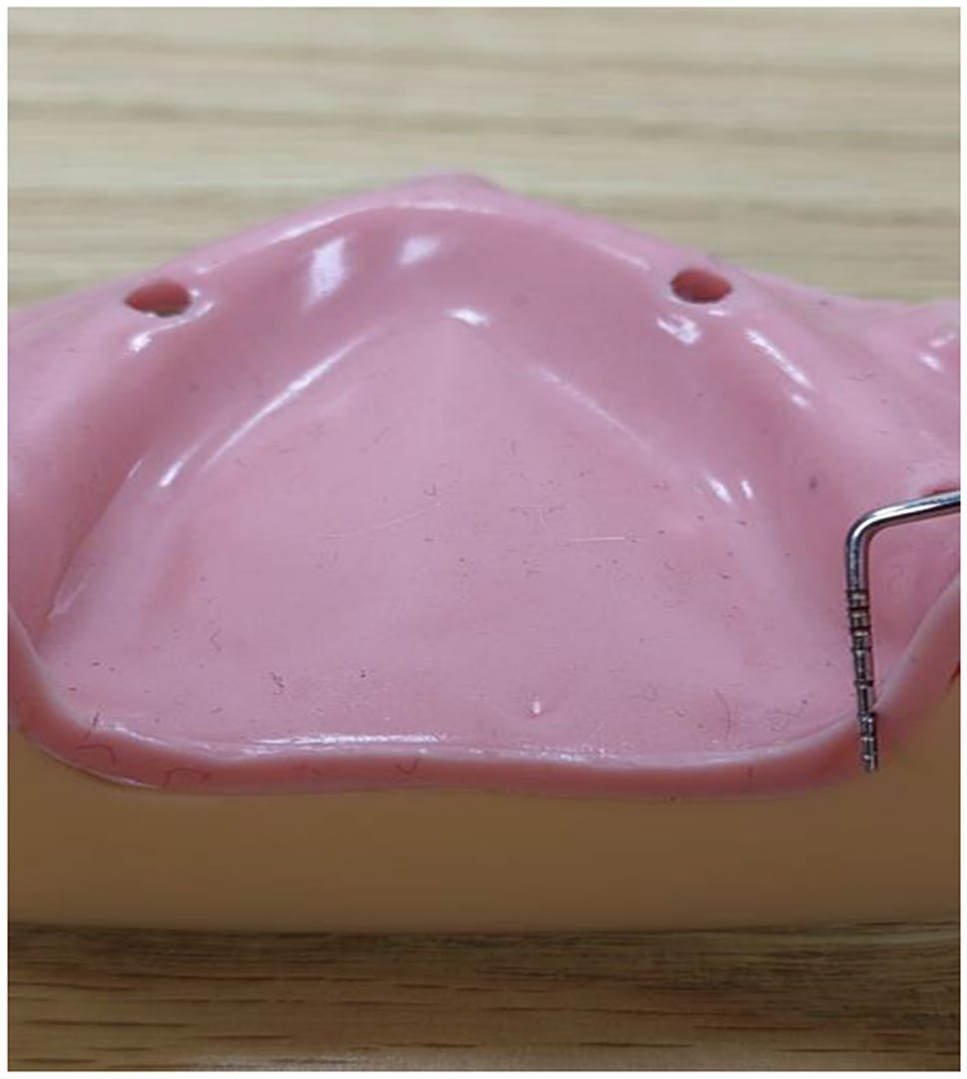
Flexible polyurethane layer on the 3D printed cast

Table 3; Fig. 6c display the results of an intraoral scan on the body’s wettability. When comparing the wettability of two situations, dry and wet, at six different distances within a plant, there was a statistically significant difference (*p* < 0.05) in mean difference and standard deviation in micrometers (see Fig. 7).

**Table 3:** Mean difference of distances in micrometer, standard deviations, *p-*value, and t-value for the effect of the wettability of intraoral scan body

	(AB)	(BC)	(CD)	(AD)	(AC)	(BD)
**Dry**	48.85 ± 0.90	24.11 ± 0.99	29.98 ± 0.59	26.29 ± 0.44	47.24 ± 0.64	44.93 ± 0.93
**Wet**	49.31 ± 0.81	24.35 ± 0.93	30.68 ± 0.86	27.13 ± 0.98	47.47 ± 0.77	45.33 ± 1.54
**T**	1.851	0.865	3.258^*^	3.850^*^	1.111	1.108
***P***	0.071	0.392	0.002^*^	0.001^*^	0.272	0.274

24 replica. Data was expressed using Mean ± SD. SD: Standard deviation. t: Student t-test. *p*: *p*-value for comparing between Dry and Wet. *: Statistically significant at *p* < 0.05

## Discussion

This in-vitro study measured variables along the x, y, and z-axes to determine the impact of saliva, various intraoral scan body exposed lengths, and material on the accuracy of the digital implant transfer by measuring six interimplant distances in mm from fixed points without superimmpostion. The study found that utilizing a long intraoral scan body resulted in improved accuracy in digital implant transfer compared to using a short one. In addition, this difference was statistically significant (*p* < 0.05). These findings are consistent with those of other studies that found a higher trueness value for the longer intraoral scan body compared to the shorter one. Among these studies, Petchmedyai et al., examined the impact of height of the scan body and the methods of alignment in CAD software on the accuracy of digital implant transfer, particularly for varying implant depths. Furthermore, stereolithography was utilized to generate three half-arch implant models, each with a different implant depth. These models were used to simulate three different scan body exposure scenarios: fully exposed, 2/3 exposed, and 1/3 exposed. It was able to replicate the scanned body image using CAD software in order to capture deficiencies and alignment approaches. The use of 3D analysis software allowed for the evaluation of the deviation of simulated implant placements acquired from various scenarios. The 1/4 upper- and lower-part scan body insufficiency utilizing the 1-point alignment method in the 1/3 exposed scan body measured the largest angular and linear deviation (0.237 ± 0.059 degrees, 0.084 ± 0.068 mm) [24]. 

According to a study by Gómez-Polo et al., digital implant transfer performed better when the scannable portion of the intraoral scan body was raised [25]. In contrast to the results of this study, Sicilia et al. found that the supramucosal height of the scan body did not have an impact on the accuracy of intraoral scans in 17 out of 18 planned comparisons [26]. However, the findings can vary depending on the specifics of the implant scan. The findings of this in-vitro study corroborated those of a study conducted by Gomez-Polo et al., which demonstrated that the accuracy and precision of intraoral scans were impacted by the surface humidity. Specifically, when the surfaces of the two different materials were moistened with artificial saliva, there was a significant difference (*p* < 0.05) between them. The in-vitro study found that titanium intraoral scan bodies exhibited significantly higher accuracy (*p* < 0.05) compared to PEEK intraoral scan bodies for both types of intraoral scan body materials [27]. This study findings align with a study conducted by Lee et al., they analyzed the differences in terms of the materials used for the scan bodies and found that titanium had a higher trueness value than PEEK. Additionally, there were significant differences between the two intraoral scan bodies in terms of the materials utilized. Titanium scan bodies exhibited a higher trueness value compared to PEEK scan bodies. This finding can be attributed to the dissimilarity in size and shape between the titanium and PEEK scan bodies. Moreover, the titanium scan bodies have two surfaces, whereas the PEEK bodies only have one flat surface [28]. Therefore, the null hypothesis was rejected. Nevertheless, the significant differences may indicate a decrease in scanning precision caused by variations in the surface’s humidity. The distances for all the six casts were measured using a coordinate measuring machine (CMM). It was used to record three dimensional coordinates (x, y and z) at the centers of implant platforms. The accuracy of the CMM was 0.0001 mm (according to manufacturer) and one operator made all measurements. The center of healing abutments which were tightened to the implant fixtures were considered the center of implant position. The center of each healing abutment was located using CMM probe by touching eight points on the circumference of the outer diameter of the healing abutment and then the center was determined. From this point, planar surface was regarded XY.

Four points on the upper surface of each healing abutment were measured to form a plane used to calculate the vertical distances between four healing abutments in the Z-axis.

The distances (in micrometers) between the implant centers with the reference point were calculated according to the following formula:

The distance from the reference point (r) =$\:\surd\:{\text{x}}^{2}+{y}^{2}+{z}^{2}$ To obtain this gold standard value with high accuracy, coordinate measuring machines (CMMs) have been introduced in dentistry [29]. CMMs are widely used in industrial applications and are known to be precise and able to be used in the dental field, which requires micro-unit accuracy. This machine contacts the desired point with a probe and records its coordinates. They recognize the shape of the object and measure dimensional length with high accuracy [30]. When a CMM is used with a reliable reference point, the gold standard value can be obtained for accuracy assessment of a digitized model with various scanning modalities.There are other alternatives for coordinate measuring machine(CMM) such as: photogrammetry and sterophotogrammetry.

### Limitations

1. The process of aligning teeth was carried out digitally using the EXOCAD design software without the use of a maxillary cast as a reference model, as the presence of the antagonist maxillary teeth makes the setting, alignment of the opposing lower teeth more accurate.
2. Limitation of digital scanning due to the absence of teeth.
3. Using of one type of intraoral scanner.
4. The difference in shape and geometery between the two different intraoral scan bodies of the same company.

## Conclusions

1. The use of a longer PEEK or TITANIUM intraoral scan body improved the accuracy of the digital implant transfer results.
2. The accuracy of digital implant transfer using an intraoral scan body improved when performed in a dry setting compared to a moist one.
3. Results of TITANIUM intraoral scan bodies were more reliable and precise compared to the those obtained from the coordinate measuring machine(CMM) than PEEK intraoral scan bodies.

## Data Availability

On reasonable request, the datasets utilized or analyzed during the present study are accessible from the corresponding author.
